# HACE1 as a bridge between oxidative stress and autophagy

**DOI:** 10.3389/fimmu.2025.1595601

**Published:** 2025-08-29

**Authors:** Jiyue Xia, Youhong Jiang, Xiangjun Xin, Ting Li, Wenbo Liao, Zhijun Xin

**Affiliations:** ^1^ Department of Orthopedic Surgery, Affiliated Hospital of Zunyi Medical University, Zunyi, Guizhou, China; ^2^ Department of Anesthesiology, Weifang Traditional Chinese Medicine Hospital, Weifang, Shandong, China; ^3^ Department of Respiratory and Critical Care Medicine, Qixingguan District People’s Hospital of Bijie, Bijie, Guizhou, China

**Keywords:** autophagy, cellular stress, HACE1, oxidative stress, ubiquitylate

## Abstract

HECT domain and ankyrin repeat-containing E3 ubiquitin-protein ligase 1 (HACE1) is a well-known tumor suppressor and is essential for embryonic development. In recent years, researchers have increasingly discovered that HACE1 plays a vital role in the pathological process of many degenerative diseases. HACE1 is regarded as a stress-responsive gene whose expression is induced by a variety of stress stimuli. The expression of HACE1 counters cell stress damage by promoting the expression of antioxidant genes and inhibiting ROS production from Rac1-dependent NADPH oxidase. Meanwhile, HACE1 serves as a crucial E3 ubiquitin ligase that activates autophagy by ubiquitinating autophagy-related receptors to clear irreversibly oxidized biomolecules within the cell. Therefore, HACE1 is essential for cellular survival by maintaining antioxidant defense mechanisms and autophagic flux. Pharmacological and genetic modulation of HACE1 expression holds potential therapeutic value in age-related diseases such as neurodegenerative disorders, cardiovascular diseases, and cancer.

## Graphical Abstract

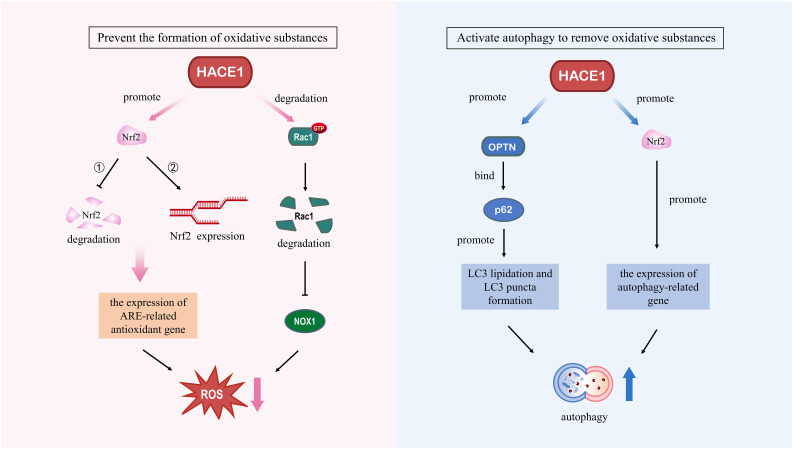

## Highlights

- HACE1 acts as a stress response factor, which activates antioxidative stress and autophagy.
- HACE1 expression is induced by physiological stimuli, while pathological stimuli deplete it.
- HACE1 is an important link between autophagy and oxidative stress.
- HACE1 plays a therapeutic role in degenerative diseases such as neurodegenerative diseases, cardiovascular diseases and cancer.

## 1 Introduction

Oxidative stress occurs when the production of reactive oxygen species (ROS) overwhelms the capacity of the body’s antioxidant defense systems, ultimately leading to cellular and tissue oxidative damage (1). ROS comprise both radical species, such as superoxide anions and hydroxyl radicals, and non-radical molecules like hydrogen peroxide and singlet oxygen (2). Under physiological conditions, these ROS molecules function as signaling entities, capable of activating autophagy and antioxidant defense mechanisms to maintain cellular homeostasis (3, 4). However, excessive extracellular stimulation leads to ROS accumulation and also depletes the endogenous antioxidant system, resulting in cellular redox imbalance, mitochondrial dysfunction, and DNA oxidative damage, which ultimately leads to oxidative damage and even cell death (5). Increasing evidence indicates that oxidative stress plays a crucial role in the pathogenesis of various age-related diseases, including neurodegenerative disorders, cardiovascular diseases, and cancer (6–8).

Autophagy is a catabolic process that maintains cellular homeostasis by degrading and recycling cellular components and damaged organelles (9). Research has identified autophagy as an effective alternative mechanism for antioxidant defense systems, alleviating cellular oxidative damage by removing irreversible oxidative products and damaged mitochondria within cells (10). It is well known that excessive ROS disrupts critical cellular components, including DNA, proteins, and lipids (11). Among these disruptions, DNA oxidative damage includes oxidative modifications of DNA bases and single-stranded or double-stranded DNA breaks (12). Oxidative damage to proteins causes protein carbonylation and the accumulation of unfolded proteins (13, 14). Lipid peroxidation, mainly caused by cell membrane or subcellular organelle membrane phospholipid and polyunsaturated fatty acid (PUFA), produces highly destructive carbonyl compounds and disrupts the integrity of cell membranes (15). Increasing evidence indicates that autophagy alleviates cellular oxidative damage by engulfing and degrading oxidized substances (16–18).

Recent research has found that *HACE1* (HECT domain and ankyrin repeat-containing E3 ubiquitin-protein ligase 1) serves as a stress-protective gene that plays a crucial role in heart diseases, neurodegenerative diseases, and tumors. As a cross-regulator of oxidative stress and autophagy, HACE1 could mitigate the production and accumulation of oxidative damage substances by enhancing the antioxidant defense system and activating autophagy. However, previous reviews have solely focused on the role and mechanisms of HACE1 in a single disease, and a better understanding of its molecular pathogenesis is required to enable the development of targeted therapies. In this review, we first introduce the protein structure and function of HACE1 and then provide a systematic mechanism analysis of its redox signaling pathway, autophagy pathway, and tumor suppressor pathways. Therefore, the role of HACE1 in the adaptive defense system of the organism makes it a promising therapeutic target in age-related degenerative diseases. Encouragingly, researchers have discovered that certain natural antioxidants can upregulate HACE1 expression, thereby maintaining cellular homeostasis by activating autophagy and enhancing antioxidant stress responses (19–21). Undoubtedly, akin to its downstream target Nrf2, HACE1 is also destined to emerge as a novel antioxidant stress factor and attract widespread attention.

## 2 Basic structure and function of HACE1

**Figure 1:**
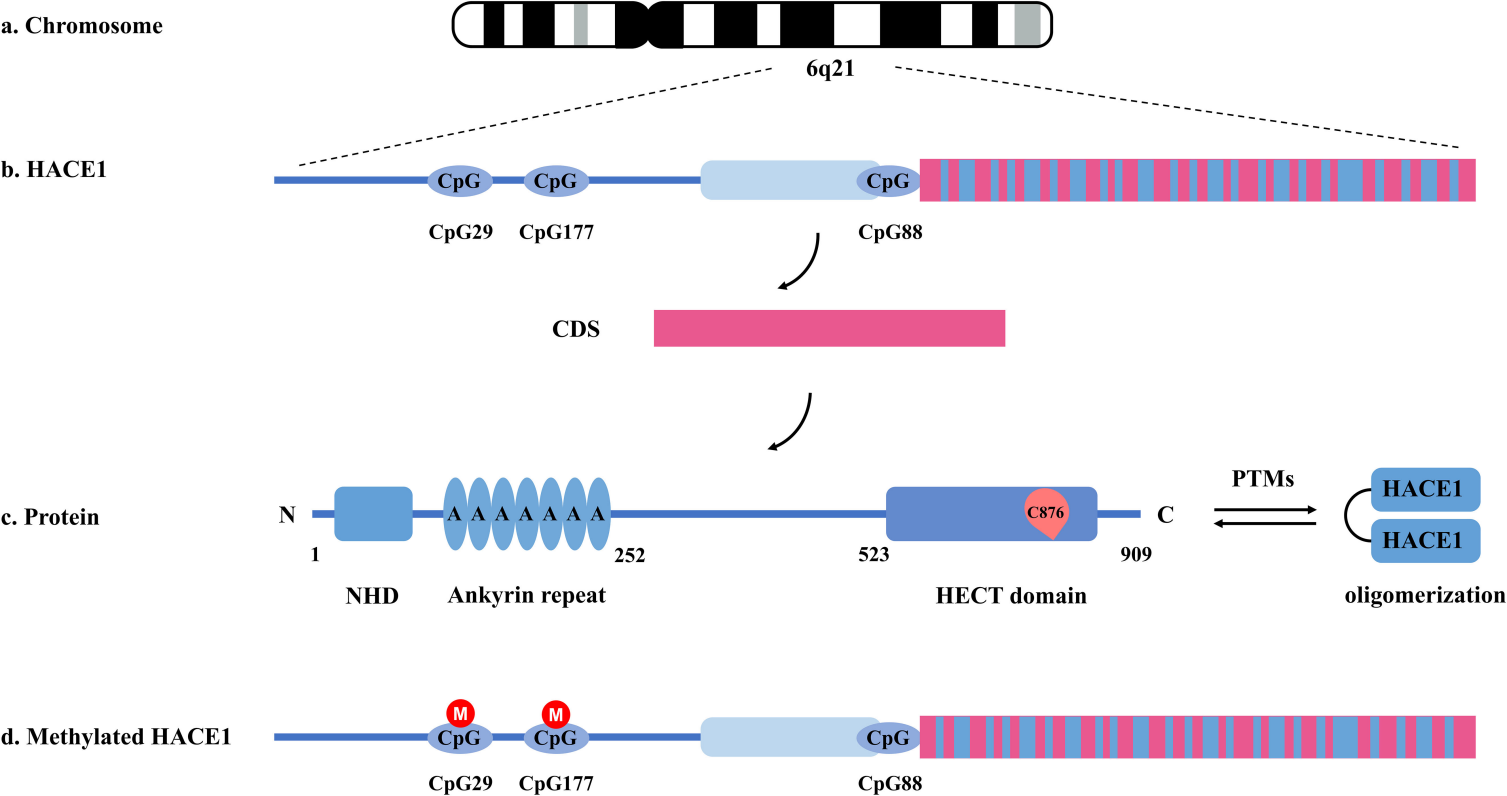
Basic structure and function of HACE1. **(a, b)** HACE1 is a tumor suppressor gene located on human chromosome 6q21 encoding a protein known as E3 ubiquitin protein ligase. **(c)** The ubiquitination ability of HACE1 protein is related to its oligomerization state and post-translational modifications. **(d)** Transcriptional activity of HACE1 is strongly correlated with CpG methylation levels in the HACE1 gene promoter.

*HACE1*, known as a renowned tumor suppressor gene, is located within the tumor suppressor region on chromosome 6q21, encoding a protein with a relative molecular mass of approximately 103 kDa (22). As shown in **Figure 1**, the HACE1 protein is composed of 909 amino acid residues and is generally localized in the Golgi apparatus and endoplasmic reticulum (22, 23). It consists of an N-terminal helical domain (NHD), seven ankyrin repeats (ANK), a linker middle domain (MID), and a conserved C-terminal catalytic HECT domain (24). The C-terminal HECT domain is essential for the E3 ubiquitin ligase activity of HACE1, and mutation of the conserved HECT domain cysteine residue 876 (HACE1 C876S) has been recognized to eliminate its E3 ubiquitin ligase activity (25). The NHD and MID domains influence the ubiquitination capacity of HACE1 by regulating its oligomerization state because the N-terminal helix of one monomer limits access to the C-terminal domain of the other monomer in the dimer (24, 26). Additionally, it was found that Group-I PAKs could affect the oligomerization state of HACE1 by mediating its post-translational modifications (PTMs), which consequently affects its ubiquitination activity (26). Therefore, we recommend further research on how to regulate PTMs of HACE1 to affect its activity. The ANK domain is responsible for determining the autophagic activity of HACE1 by mediating protein-protein interactions (27). In summary, every domain of HACE1 is essential for its function.

**Figure 2:**
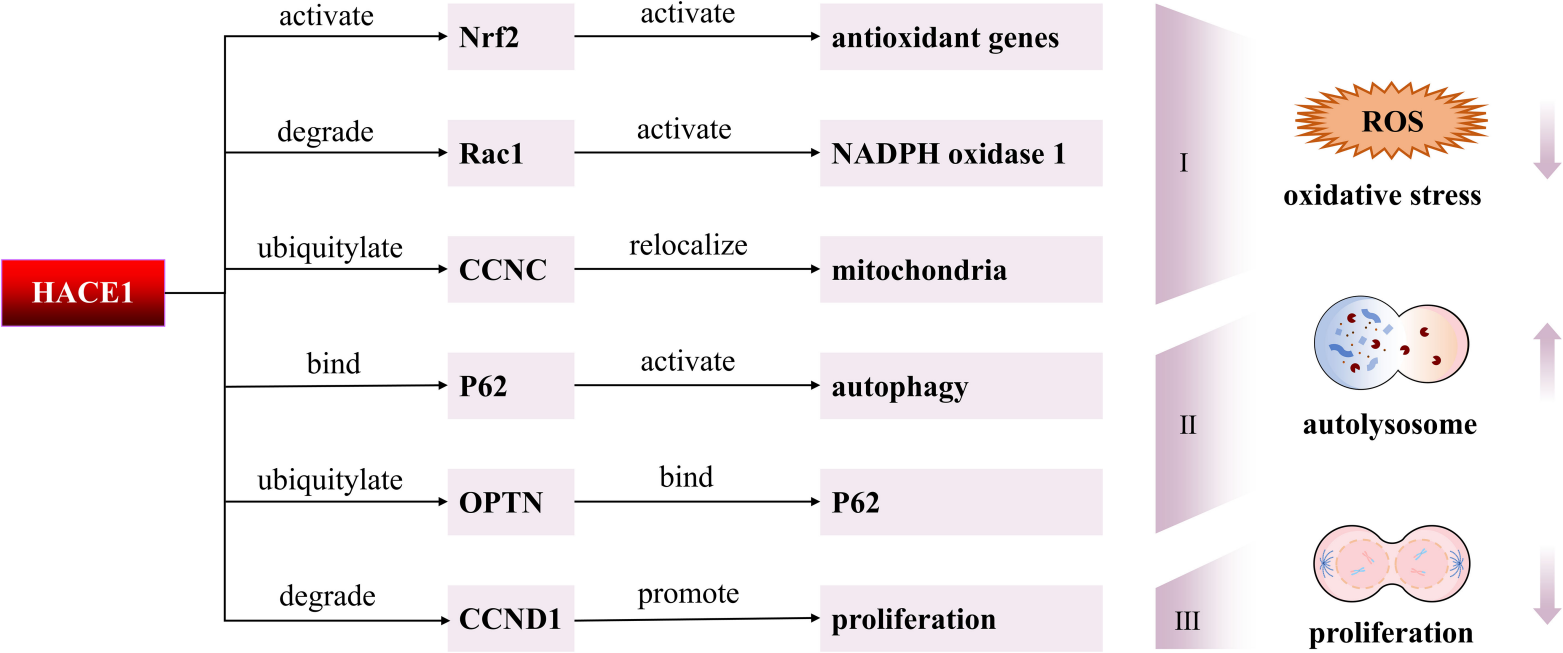
Molecular mechanisms of HACE1 in degenerative diseases. First, HACE1 might reduce oxidative stress by modulating Nrf2, Rac1, and CCNC. Second, HACE1 activates autophagy by ubiquitinating OPTN to promote the interaction of OPTN with p62. Moreover, HACE1 can interact directly with p62 to activate autophagy. Third, HACE1 inhibits tumor cell proliferation by suppressing the expression of CCND1.

HACE1 is a bifunctional E3 ubiquitin ligase. On one hand, HACE1 transfers ubiquitin to target substrates via its HECT domain E3 ubiquitin ligase activity, leading to subsequent proteasomal degradation. Studies have shown that the tumor suppressor function of HACE1 is closely linked to its E3 ubiquitin ligase activity. Mechanistically, HACE1 mediates the ubiquitination and degradation of Rac1 via its E3 ubiquitin ligase activity, thereby inhibiting ROS generation from Rac1-dependent NADPH oxidase and cellular hyperproliferation induced by cyclin D1 (28–30). While the activity and level of Rac1 can be modulated by regulating the ubiquitination capacity of HACE1 (24, 26, 31). On the other hand, HACE1 can activate autophagy independently of its E3 ubiquitin ligase activity. Research indicates that HACE1 activates p62-dependent selective autophagy through protein-protein interactions mediated by its ANK domain (27). Furthermore, HACE1 is capable of interacting with OPTN without relying on the E3 ubiquitin ligase activity (32). In addition, HACE1 can regulate the ubiquitination and degradation efficiency of target proteins by influencing the protein-protein interactions through the ANK domain (31). For example, HACE1 controls the level of active Rac1 and cell migration by interacting with Rac1 through the ANK domain (33). All in all, HACE1 is a critical E3 ubiquitin ligase and exerts its biological function through the activation of either the proteasome pathway or the autophagy lysosomal pathway, as shown in **Figure 2**.

## 3 HACE1 dysfunction plays a significant role in neurodegenerative diseases

Neurodegenerative diseases are a class of clinical syndromes characterized by progressive and irreversible damage to specific neurons, leading to the gradual decline of corresponding neurological functions (34). As shown in **Table 1**, their common pathological mechanism lies in the misfolding and aggregation of intracellular neuronal proteins (such as Aβ, tau, and α-synuclein), forming insoluble deposits that induce oxidative stress and mitochondrial dysfunction, ultimately leading to synaptic dysfunction and neuronal cell death (35). As a novel molecular target in neurodegenerative diseases, HACE1 is closely related to neurodegenerative diseases such as Alzheimer’s disease, Parkinson’s disease and Huntington’s disease (36). How HACE1 deficiency leads to neurodegenerative proteinopathies is not fully understood, but two distinct mechanisms are thought to be particularly relevant. First, HACE1 deficiency causes disruption of the ubiquitin-proteasome system and autophagolysosome pathway, resulting in protein aggregates that cannot be cleared (27). Second, HACE1 deficiency causes dysfunction of mitochondria and antioxidant defense systems, resulting in increased oxidative stress and protein misfolding, which exacerbates protein aggregate formation (37, 38).

**Table 1:** The formation of misfolded protein aggregates is a hallmark of neurodegenerative diseases.

Neurodegenerative disease	Functional protein	Protein aggregates	Proteinopathy
Alzheimer's disease (AD)	Amyloid beta (Aβ); Tau	Amyloid plaques; Neurofibrillary tangles (NFTs)	Amyloidopathy Tauopathy
Parkinson's disease (PD)	α-synuclein	Lewy bodies (LBs)	Synucleinopathy
Huntington's disease (HD)	Mutant huntingtin (mHTT)	mHTT aggregates (polyQ aggregates)	Polyglutamine disease (polyQ disease)

## 4 HACE1 expression promotes cellular antioxidant defense capacity

*HACE1* functions as a ubiquitous early stress response gene induced by various stress stimuli, including endoplasmic reticulum stress, virus infection, nutrient deprivation, hypoxia and gamma irradiation (39, 40). The role of HACE1 in ischemia reperfusion (IR) injury has recently been reported. IR for a short period of time could upregulate HACE1 expression and activate the antioxidant stress pathway to alleviate IR-induced cardiac injury (41). However, prolonged IR stimulation resulted in depletion of HACE1 reserves, which exacerbated oxidative stress and inflammation in neurons and cardiomyocytes (42, 43). It is well known that oxidative stress is the main mechanism of tissue damage induced by IR (44). Thus, HACE1 expression induced by this hemodynamic stress is a compensatory response of the body, and lack of HACE1 results in cell sensitivity to oxidative damage and dependence on extracellular nutrients (45, 46). A growing body of research suggests that HACE1 plays an important role in regulating oxidative stress in age-related degenerative diseases.

### 4.1 HACE1 protects against oxidative damage by activating Nrf2

Nuclear factor erythroid-derived 2-like-2 (Nrf2), a key antioxidant transcription factor, plays an important role in enhancing cellular resistance to toxic stimuli and oxidative stress damage (47). Under resting conditions, Keap1 functions as a substrate adaptor protein for a Cul3-dependent E3 ubiquitin ligase complex. Within the E3 ubiquitin ligase complex based on the Cul3 scaffold, Keap1 forms homodimers through its BTB domain, which allows it to specifically recognize and expedite the ubiquitination and degradation of cytoplasmic Nrf2 (48). The oxidative modification of specific cysteine residues on Keap1 causes spatial conformational changes that cause Nrf2 to separate from ubiquitination (49). Furthermore, Nrf2 expression and nuclear translocation can be induced by oxidative stress, which eventually results in Nrf2 accumulation in the nucleus (50). Binding to small Maf proteins, nuclear Nrf2 activates antioxidant response elements (AREs) and triggers the expression of several genes related to detoxification and antioxidants (51).

HACE1 is an oxidative stress response factor that is essential for maintaining cellular antioxidant defense by mediating Nrf2 activation. Specifically, HACE1 prevents Nrf2 ubiquitination and proteasomal degradation by competing with Keap1, thereby stabilizing Nrf2 protein and enhancing its transcriptional activity in response to oxidative stress (52). This process requires HACE1 to have an intact domain, because the loss of either the HECT or ANK domain will lead to the disruption of Nrf2 protein homeostasis (52). Hence, the cytoprotective effect of HACE1 may be mainly attributed to the antioxidant activity of Nrf2. Rotblat et al. (45) found that HACE1 is closely associated with neurodegenerative diseases because its expression levels are reduced in the striatum of Huntington’s disease patients. Furthermore, knockout of HACE1 resulted in impaired antioxidant stress response in mice, and this sensitivity to oxidative stress could be restored by ectopic expression of Nrf2 (45). In addition to inhibiting neurodegeneration, HACE1 has been reported to activate the Nrf2/ARE signaling pathway, upregulate HO-1 and NQO1 antioxidant genes, and attenuate H/R-induced oxidative stress and inflammation, making it a promising therapeutic target and predictor of cardiac disease (42). Consistent with findings in the nervous system, the protective effect of HACE1 in the heart was also confirmed in Nrf2-mediated responses (53).

### 4.2 HACE1 alleviates oxidative damage through ubiquitination and degradation of active Rac1

Ras-related C3 botulinum toxin substrate 1 (Rac1) is a small GTPase belonging to the Rho GTPase family and an essential intracellular signaling molecule (54). As a signal transduction molecular switch, Rac1 alternates between inactive and active states to transduce cellular signals. The activated form of Rac1 is an indispensable subunit of NOX, which is essential for the subsequent activation of NOX and ROS generation (55). NOX is a transmembrane protein composed of two catalytic subunits (gp91phox and p22phox) and four regulatory subunits (p47phox, p40phox, p67phox and Rac1) (56). In a resting cellular state, NOX is inactive because there are no cytoplasmic subunits bound to the cell membrane (57). When cells are stimulated by internal and external factors, the cytoplasmic subunit of NOX is modified by phosphorylation to undergo a conformational change and ultimately assembles with the membrane-bound subunit to form a catalytically active oxidase (58). In addition, previous study has reported that there is a mutual activation between Rac1 and NOX, which is instrumental in sustaining physiological levels of ROS required for axonal growth of hippocampal neurons (59).

**Figure 3:**
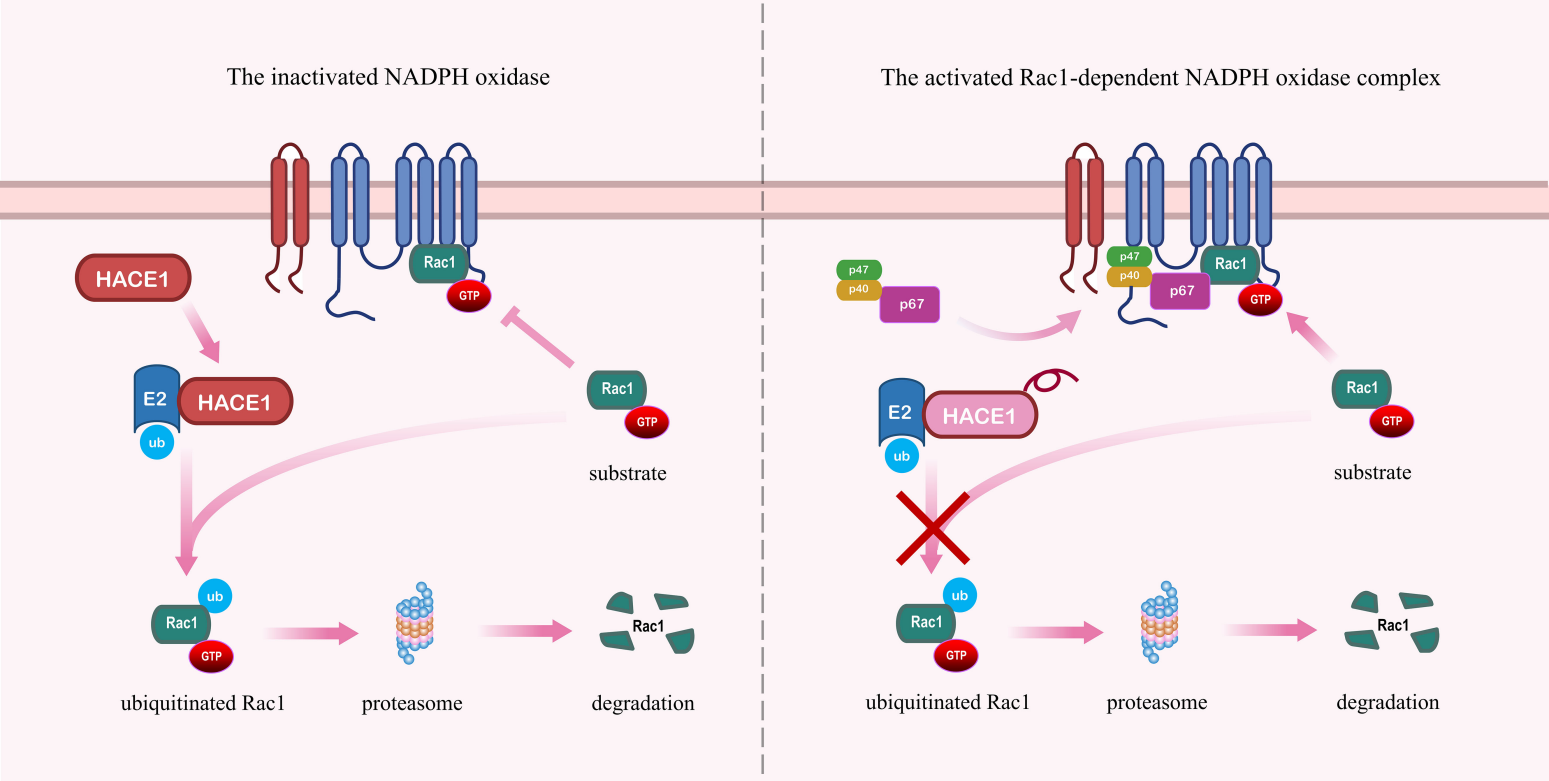
HACE1 prevents the formation of the NADPH oxidase complex by promoting the ubiquitination and degradation of Rac1. Left panel: HACE1 inhibits NADPH oxidase by ubiquitinating and degrading activated Rac1. Right panel: Deficiency of HACE1 impedes the ubiquitination and degradation of activated Rac1, thereby exacerbating the formation of the NADPH oxidase complex.

The current study has found that the E3 ubiquitin ligases HACE1 and the IAPs are responsible for the ubiquitination and proteasome degradation of activated Rac1 (60). During the further identification process, Stéphanie et al. (61) have found that HACE1 is the E3 ubiquitin ligase with the greatest influence on the regulation of Rac1 turnover and activity. As shown in the **Figure 3**, it has been shown that HACE1 controls the activity of the Rac1-dependent NOX complex by targeting Rac1 for degradation, which inhibits the production of superoxide and ROS (29, 62). The deficiency of HACE1 leads to the hyperactivation of Rac1, resulting in increased cellular malignancy and invasiveness, thereby promoting cancer progression (25, 55, 63). In addition to its therapeutic role in tumors, HACE1 is significant for embryonic development through the Rac1 signaling pathway (64). Lack of HACE1 causes the accumulation of Rac1 and NADPH oxidase-dependent ROS production, which ultimately results in vertebrate cardiac and neurological hypoplasia (65, 66). Zang et al. (67) have found that HACE1 expression decreased with age in mouse models of Parkinson’s disease. HACE1 knockdown exacerbates neuroinflammation and symptoms in PD in a Rac1-dependent NADPH oxidase manner (67). Therefore, targeting HACE1 to inhibit the Rac1 signaling pathway to alleviate cellular oxidative stress would be an effective therapeutic strategy in degenerative diseases.

As research progressed, it has been discovered that activation of Rac1 is necessary for NADPH oxidase-dependent ROS production (62). Thus, one might wonder whether HACE1 regulates oxidative stress through degrading Rac1. To confirm this conjecture, we summarize the functional mechanisms and actions of HACE1 in regulating Rac1 in **Table 2**. The expression of HACE1 protein is influenced by various factors, including gene editing, HACE1 mutations, and the regulation of HACE1-PTMs. HIF inhibitors (FIH) have been found to inhibit the ability of HACE1 to ubiquitinate Rac1 by hydroxylating HACE1 at residue N191 of the ANK domain (68). Neuroepithelial transforming gene 1 (NET1) protects Rac1 from degradation by HACE1 and is essential for actin dynamics and meiotic spindle formation (69). Furthermore, HACE1-driven Rac1 ubiquitination degradation also requires another E3 ubiquitin ligase, TRIP12. Silencing TRIP12 blocks the inhibitory effect of HACE1 on Rac1 protein degradation and esophageal cancer (ESCA) tumor growth (70). Rac1 has been considered a central signaling hub essential for many oncogene-induced transformations, playing a crucial role in cancer cell proliferation, migration, and invasion (71). HACE1 inhibits the progression of a variety of tumors, including breast cancer, lung cancer, prostate cancer, and osteosarcoma, by regulating the ubiquitination and proteasomal degradation of Rac1. Specifically, HACE1 protects cells from oxidative stress-induced DNA damage and cycle D1-driven hyperproliferation by blocking ROS production through Rac1-dependent NADPH oxidase. In addition, HACE1 plays a crucial role in the growth and development of the organism, especially the heart and nervous system. Taken together, HACE1 regulates cellular oxidative stress in a Rac1-dependent manner and is essential in organ development and tumor suppression.

**Table 2:** Functional mechanisms and effects of HACE1 ubiquitination degradation activity Rac1.

HACE1	Target protein	Inducer	Functional mechanisms	Effects	References
Gene editing	Active Rac1	CNF1	HACE1 Controls CNF1-Induced Degradation of Rac1	HACE1 depletion dramatically reduced endothelial cell monolayer invasion by pathogenic bacteria	(61)
Gene editing	Active Rac1	HGF	HACE1 promotes the ubiquitylation and degradation of the active form of Rac1 in response to growth factor signaling	HACE1 controls cell migration by regulating Rac1 degradation	(33)
Gene editing	Active Rac1	H2O2	HACE1 regulates multiple Rac1-dependent NADPH oxidases by targeting Rac1 for degradation	HACE1 protects cells from ROS-induced DNA damage and cyclin D1-mediated hyperproliferation.	(29)
Gene editing	Rac1-dependent NADPH oxidase complexes	Gln starvation	HACE1 expression alleviates Gln starvation-induced ROS elevation and cell death	HACE1 depletion leads to Gln starvation-induced ROS elevation and cell death(HACE1 depletion causes cellular fate to dependence on nutrients)	(46)
Gene editing	Active Rac1	EGF/HRG	HACE1 controls EGF/HRG-induced Rac1 activation and mammary epithelial cell transformation	HACE1 deficiency combined with HER2 overexpression leads to Rac1 hyperactivation, resulting in breast cancer transformation and progression	(63)
Gene editing	Active Rac1	/	The defects caused by the HACE1 knockdown are partly rescued by knockdown of Rac1	HACE1 depletion in Xenopus laevis embryos leads to diverse developmental defects, including a shortened body axis and the inhibition of eye and pigment formation	(64)
HACE1 mutations	Active Rac1	/	Mutations in HACE1 ANK and MID domains that modulate its ubiquitylation activity on Rac1	Missense mutations in HACE1 impair the ubiquitination of Rac1 and cell growth control	(31)
Gene editing	Active Rac1	/	HACE1 blocks *de novo* generation of ROS by Rac1-dependent NADPH oxidase	HACE1 is critical to the normal development and proper function of the vertebrate heart via a ROS-dependent mechanism.	(65)
HACE1-PTMs	Active Rac1	CNF1/VEGF	CNF1/VEGF treatment promotes the phosphorylation of HACE1 on Ser-385 in a Rac1-dependent manner	A feedback inhibition of HACE1 ubiquitination activity on Rac1 by group-I PAK kinases	(26)
Gene editing	Active Rac1	/	HACE1 downregulation leads to increased Rac1 activation and ROS accumulation, which promotes osteosarcoma progression	HACE1 inhibits the growth, invasion, and metastasis of osteosarcoma cells *in vitro* and *in vivo*	(25)
HACE1-PTMs	Active Rac1	Hypoxia	Hypoxia-induced FIH inactivation inhibits HACE1 hydroxylation to promote HACE1-dependent Rac1 degradation	HACE1 knockdown accelerates hypoxia-induced breast cancer cell migration and invasion	(68)
Gene editing	Active Rac1	/	HACE1 KO mouse brains and SPPRS patient-derived fibroblasts have elevated Rac1 and ROS levels	HACE1 KO mice exhibit the clinical features of SPPRS including neurodevelopmental disorders	(66)
Gene editing	Active Rac1	KRAS G12D	Ablation of Rac1 and Rac2 completely avoids KRas G12D-driven lung tumor progression in HACE1-deficient mice	HACE1 prevents lung carcinogenesis via inhibition of Rac-family GTPases (RAC1 and RAC2)	(55)
Gene editing	Rac1	KRAS G12D	HACE1 inhibits HIF1α accumulation under hypoxic conditions in a Rac1-dependent manner	HACE1 deficiency leads to HIF1α accumulation in KRas G12D-driven lung tumors	(39)
Gene editing	Active Rac1	DHT	The combination of Rac1 inhibitors and AR antagonists suppresses AR gene expression in androgen-sensitive prostate cancer cells.	HACE1 deletion contributes to prostate cancer progression by causing hyperactivation of the Rac1 signaling pathway	(72)
Gene editing	Active Rac1	LPS/MPTP	HACE1 alleviates LPS/MPTP-induced neuroinflammation in PD via the Rac1-NADPH oxidase pathway	HACE1 negatively regulates neuroinflammation through ubiquitylating and degrading Rac1 in Parkinson’s disease models	(67)
Gene editing	Active Rac1	KRAS G12D	HACE1 destabilizes mTOR by targeting Rac1 within mTOR-associated complexes	Silencing Rac1 in HACE1-deficient mice reverses enhanced mTOR expression in KRas G12D-driven lung tumors	(73)
NET1	Active Rac1	/	NET1 protects RAC1 from HACE1-mediated degradation	Ectopic RAC1 expression in Net1-depleted oocytes rescues meiotic defects	(69)
Gene editing	Active Rac1	/	HACE1 and TRIP12 combine to regulate the ubiquitinated degradation of RAC1 proteins	HACE1 inhibited tumor growth of esophageal cancer (ESCA) by degrading Rac1	(70)

## 5 HACE1 activates cellular autophagy through ubiquitination of autophagy-related receptor proteins

Autophagy is a catabolic degradation process necessary for cell homeostasis, which is an extremely important alternative pathway for cellular antioxidants (74). When autophagy is impaired, it leads to mitochondrial dysfunction, excessive ROS accumulation and disruption of cell homeostasis (75). Viedma-Poyatos et al. (76) discovered a vicious cycle of mutual intensification between oxidative stress and protein aggregation, resulting in abnormal intracellular protein aggregation and cytotoxicity. Accumulating evidence suggests that protein aggregation and mitochondrial dysfunction are common features associated with many age-related degenerative diseases, especially neurodegenerative diseases, cardiovascular diseases and tumors (77–79). In these diseases, autophagy efficiency decreases in various ways with age. Autophagy is essential for maintaining cell survival under oxidative stress conditions by selectively removing damaged organelles and protein aggregates (80). Therefore, the role of HACE1 gene in antioxidant stress has recently attracted increasing attention. Patients with mutations in the *HACE1* gene contribute to severe neurodevelopmental deficits by causing autophagy defects and oxidative stress (37). As an autophagy adapter protein, HACE1 prevents cellular oxidative damage and tumor progression by activating autophagy to remove toxic proteins and damaged organelles through the ubiquitination of the selective autophagy receptors p62 and OPTN.

### 5.1 HACE1 activates selective autophagy through ubiquitination of OPTN

Optineurin (OPTN) is a multifunctional protein with multiple domains and is broadly expressed in many organs and tissues in the human body (81). OPTN consists of two coiled-coil (CC) domains, a leucine zipper (LZ) domain, an LC3-interaction region (LIR), a HACE1-interaction region (HIR), a ubiquitin-binding (UBAN) domain, and a C-terminal Npl4-type zinc finger (NZF) domain (82). The N-terminal CC domain of OPTN binds directly to the C-terminal domain of TBK1, forming stable OPTN-TBK1 heterotetrameric complexes that are recruited into ubiquitinated aggregates (83). The LZ domain of OPTN interacts with ATG9A, which facilitates the recruitment of ATG9A vesicles for autophagosome formation (84). The LZ and LIR domains of OPTN bind to the N-terminal death effector domains (DEDs) of caspase 8 and regulate TNF-α-induced apoptosis (82). In addition, Wauters et al. have discovered that OPTN binds to ubiquitinated mitochondria through its UBAN domain and recruits LC3 to wrap damaged mitochondria to form autophagosomes through its LIR domain (85). Finally, Liu et al. have found that HACE1 can interact with OPTN through its N-terminal ANK structural domain, and the region on OPTN that interacts with HACE1 is defined as the HACE1 interaction region (HIR) (32).

Previous studies have established that TANK-binding kinase 1 (TBK1) induces autophagy through phosphorylation of the autophagy receptors p62 and OPTN (86). TBK1 phosphorylates directly OPTN and promotes its interaction with LC3 to accelerate autophagy efficiency (86). In turn, OPTN enhances the phosphorylation ability of TBK1 by promoting TBK1 activation (87). This process of autophagy activation forms a positive feedback loop to increase autophagic flux. It has been shown that HACE1 is upstream of TBK1 and can inhibit virus-triggered type I IFN signaling by disrupting the formation of the MAVS-TRAF3 complex (88). Therefore, in addition to focusing on the direct interaction of HACE1 with OPTN, HACE1 may also be involved in regulating autophagy efficiency by affecting TBK1 activity.

OPTN is a recognized selective autophagy receptor that mediates interactions between ubiquitinated proteins and autophagy machinery, and lack of OPTN leads to impairment of the autophagy pathway and accumulation of oxidized proteins (32). Furthermore, the autophagy activity of OPTN is affected by its expression level as well as post-translational modifications (89). HACE1 was found to enhance the interaction between OPTN and p62 by promoting ubiquitination of OPTN, and promote the formation of autophagy receptor complex to accelerate autophagy flux (32). Here, E3 ubiquitin ligase activity of HACE1 and subsequent ubiquitination of OPTN (specifically ubiquitination at Lys193) are essential for activation of autophagy. Disruption of the polyubiquitin chain on OPTN leads to impaired formation of the p62-OPTN complex, resulting in disruption of the HACE1-OPTN-p62 autophagy axis. More and more studies show that that HACE1 activates selective autophagy mediated by the HACE1-OPTN-P62 axis through ubiquitination of OPTN, thereby inhibiting tumor cell growth and progression (20, 90, 91). Thus, HACE1 bridges the gap between the ubiquitination and autophagy pathways by ubiquitinating the selective autophagy receptor OPTN. Targeting HACE1 to induce the ubiquitination of OPTN, thereby activating cellular autophagy, holds promise for the treatment of diseases characterized by autophagy deficiency.

### 5.2 HACE1 alleviates cellular damage by activating autophagy dependent on p62

p62 (also known as sequestosome-1, SQSTM1) is an adapter protein with multiple functional domains, including N-terminal PB1 domain, ZZ domain, TB domain, nuclear localization signal, nuclear export signal, PEST domain, LIR domain, KIR domain and C-terminal UBA domain (92). The LIR and UBA domains are key regulatory elements of p62 as a selective autophagy adaptor protein. p62 directly binds to LC3-II via the LIR domain to mediate autophagic degradation of specifically ubiquitylated cargoes bound to the UBA domain of p62 (93). The efficiency of p62-mediated autophagy is not only influenced by its UBA domain and ubiquitinated cargo but also by oligomerization mediated by the PB1 domain (94). p62 regulates the recruitment of target cargo and the formation of autophagosomes through the oligomerization mediated by the PB1 domain (95). The KIR domain of p62 participates in the regulation of cellular oxidative stress by binding to Keap1 and promoting the autophagic degradation of it (96). Besides, the linker region between the PB1 and ZZ domains is known as the regulatory linker (RL) region, which acts as a regulatory switch for p62 activity by interacting with the ZZ domain to regulate the binding ability of the ZZ domain (97). Taken together, these findings indicate that p62 interacts with various cellular signaling molecules through its multiple domains, thereby executing diverse cellular functions.

Autophagy is a dynamic process including the formation of autophagosomes, fusion of lysosomes and autophagosomes, and degradation of autophagosomes by lysosomes (98). Prior research has demonstrated that the autophagic activity of HACE1 is dependent on p62. The ablation of p62 disrupted the HACE1-OPTN autophagy axis, which could be reactivated by reintroducing p62 (32). Furthermore, this study also found that the HACE1-OPTN-p62 axis activates autophagy by promoting LC3II lipidation and the formation of LC3 puncta, thereby clearing p62 and carbonylated proteins to reduce ROS production (32). This avoidance of the production and accumulation of oxidative damage may be the underlying mechanism of the HACE1-OPTN-p62 axis inhibiting tumor growth and tumorigenicity. When the heart experiences hemodynamic stress, HACE1 exerts cardioprotective effects by activating p62-dependent selective autophagy to clear ubiquitinated protein aggregates. In contrast, mice lacking HACE1 have impaired autophagic flow and are less able to resist pressure stress, leading to accelerated heart failure and increased mortality (27). Mechanistically, HACE1 directly interacts with p62 to affect the cellular level of p62 independently of its E3 ubiquitin ligase activity (27). These findings have identified HACE1 as a regulator of autophagy, which activates cellular autophagy by promoting the activation of p62, thereby mitigating cellular stress injury.

## 6 HACE1 regulates cell proliferation and oxidative stress through ubiquitinated cell cycle proteins

HACE1 is an important tumor suppressor that is depleted in many malignant tumors (63). The current study indicates that the tumor suppressor function of HACE1 may be closely related to its anti-oxidative stress capacity and autophagic activity. It has been confirmed that excessive ROS can cause oxidative damage to DNA and gene mutations, leading to cell transformation and carcinogenesis (99). HACE1 blocks the generation of ROS by Rac1-dependent NADPH oxidases, thereby alleviating the initiation and progression of tumor cells caused by DNA oxidative damage and cyclin D1-driven hyperproliferation (29). In addition, the potential therapeutic role of HACE1-activated selective autophagy in tumors has been previously discussed. Remarkably, cyclin D1 has been identified as an oncogene and is overexpressed in a variety of malignant tumors (100). Furthermore, the protein level of cyclin D1 is closely related to selective autophagy, which inhibits tumor cell proliferation through autophagy‐selective degradation of cyclin D1 (101). Therefore, these findings indicate that cyclin may be involved in HACE1 regulation of oxidative stress and autophagy that plays an important role in tumors.

### 6.1 HACE1 inhibits tumor cell growth, invasion, and metastasis through the degradation of cyclin D1

Cyclin D1 (CCND1) is a protein encoded by CCND1 gene located on chromosome 11q13 and involved in regulating cell cycle progression (102). During the process of cell proliferation, cyclin D1 activates and binds to cyclin-dependent kinase 4 and 6 (CDK4/6) to form the cyclin D1-CDK4/6 complex (103). This complex phosphorylates retinoblastoma (Rb) protein, leading to the release of the E2F transcription factor, which promotes gene expression associated with G1/S phase transition (104). However, overexpression of cyclin D1 leads to cell cycle checkpoint failure and CDK dysregulation, which promotes tumor initiation and progression (105). Furthermore, the current studies demonstrate that cyclin D1 can also recruit methyltransferase G9a independently of CDK kinase activity to induce H3K9 demethylation, thereby regulating gene expression and signaling (106).

Cyclin D1 acts as a signal transduction factor in response to various intracellular and extracellular signaling factors. For example, extracellular growth factors induce Cyclin D1 expression by activating the Ras/Raf/MAPK signaling pathway (107). Recent studies have shown that the degradation of cyclin D1 can be regulated by proteasomal and autophagic lysosomes, and GSK3β-induced T286 phosphorylation of cyclin D1 is considered to be the main mechanism of cyclin D1 proteasomal degradation (108). Activation of the PI3K/AKT signaling pathway promotes Ser9 phosphorylation of GSK3β, which leads to GSK3β inactivation and cyclin D1 stabilization (109). Additionally, cytoplasmic membrane-associated cyclin D1 augmented the phosphorylation of AKT1 at Ser473 (110). This creates the positive feedback loop required to maintain intracellular cyclin D1 levels. AMBRA1 (autophagy and beclin 1 regulator 1) is a major regulator of cyclin D1 and mediates ubiquitination and proteasomal degradation of cyclin D1 (111). It has been reported that ubiquitinated cyclin D1 can be degraded via p62-mediated selective autophagy (101).

As a well-known tumor suppressor, HACE1 can inhibit the growth, invasion, and metastasis of multiple cancer types. Penninger and colleagues have found that the tumor suppressor effect of HACE1 may be attributed to cyclin D1, and HACE1 prevents cellular stress-induced tumor cell proliferation and adhesion-dependent growth through the degradation of cyclin D1 (30). Meanwhile, they have also shown that HACE1 inhibits ROS *de novo* synthesis and cyclin D1 expression by blocking Rac1-dependent NOX activity (29). Although there are no studies to show that HACE1 directly targets cyclin D1 for degradation, cyclin D1 transcription requires Rac1 and NOX activities to complete (112). Lack of HACE1 results in increased Rac1-dependent cyclin D1 transcription (29). Moreover, the expression level of cyclin D1 is inhibited by OPTN, a substrate protein of HACE1, which did this by limiting Rac1 activation (113). Taken together, these studies support the notion that HACE1 exerts a tumor suppressor function by regulating cyclin D1.

### 6.2 HACE1 regulates mitochondrial oxidative stress and apoptosis through ubiquitination of cyclin C

Cyclin C (CCNC) is an important cell cycle protein that regulates transcription and forms the kinase module of the mediator complex with the partner kinase CDK8 and the two auxiliary subunits Med12 and Med13 (114). The mediator complex stimulates the assembly of a pre-initiation complex (PIC) and recruitment of RNA Polymerase II, which is responsible for the transcription of all protein-coding genes and most non-coding RNA genes (115, 116). Apart from transcriptional function, cyclin C is involved in the regulation of oxidative stress by activating the mitochondria-dependent cell death pathway (117). Loss of cyclin C blocks the stress-induced mitochondrial apoptosis pathway, rendering malignant tumor cells insensitive to chemotherapy (118). Recent studies report that subcellular localization of cyclin C is critical for regulating cell fate (119). Cyclin C is primarily located in the nucleus during the resting state, interacting with the Med13 to regulate transcription (120). Under oxidative stress, cyclin C translocates from the nucleus to the cytoplasm, leading to activation of the mitochondrial apoptotic pathway (121). These findings suggest that subcellular localization of cyclin C is essential for oxidative stress-induced apoptosis.

The subcellular localization of cyclin C is closely related to its protein post-translational modification. Research suggests that the phosphorylation of cyclin C is essential for its translocation from the cytoplasm to the nucleus. Oxidative stress activates Slt2p to phosphorylate cyclin C protein at Ser266 triggering the mitochondrial intrinsic pathway of apoptosis (122). HACE1 mediates the non-proteolytic ubiquitination of cyclin C in the cytoplasm, thereby promoting nuclear–mitochondrial translocation of cyclin C and maintaining the chemosensitivity of gastric cancer cells to cisplatin (118). However, it has also been shown that cyclin C relocalizes for cytoplasmic degradation in response to oxidative stress, thereby inducing normal stress-responsive gene expression (123). This apparent contradiction could be explained by the differential stages of oxidative stress processes in different cell types. However, further research is needed to explore these speculative mechanisms. In conclusion, these studies suggest that cyclin C is an important regulator of cell fate determination by regulating oxidative stress-induced mitochondrial apoptosis.

## 7 Modifications influencing HACE1 expression

As an important factor in the modulation of cellular stability and survival, HACE1 plays a significant role in regulating oxidative stress, autophagy, and tumor suppression. Researches have found that HACE1 is widely expressed in a variety of normal human tissues, with particularly abundant expression in the heart, kidney, and brain (22). Recently, an increasing number of studies have indicated that HACE1 inactivation is strongly associated with poor prognosis in neurodegenerative diseases, cardiovascular diseases, and tumors. Thus, targeting HACE1 is a promising strategy for the treatment of age-related diseases, and the specific agonists that promote HACE1 expression and the precise molecular mechanisms will be the focus of future research.

### 7.1 Methylation of HACE1

*HACE1* is an indispensable tumor suppressor gene, and hypermethylation of its gene region has been found in a variety of human malignancies, including gastric cancer, colorectal cancer, liver cancer, Wilms tumor, B-cell lymphomagenesis (124). DNA methylation of CpG islands in the promoter is an important mechanism for silencing gene expression. Under normal physiological circumstances, most of the genome is methylated, and CpG islands commonly near promoters remain typically unmethylated (125). During the process of tumorigenesis, human cancer cells undergo CpG island promoter hypermethylation and loss of non-CpG island promoter CpG methylation, resulting in cancer-specific methylation patterns (126). This epigenetic alteration affects tumor suppressor genes and oncogene expression, which may lead to the malignant progression of tumors. Previous research has shown that inactivation of *HACE1* renders mice more susceptible to spontaneous and carcinogen-induced tumors (30). The inactivation of *HACE1* may be attributed to gene silencing due to hypermethylation of CpG islands on its promoter region (124). Analysis of the TCGA database data further has confirmed that hypermethylation of CpG islands in the *HACE1* promoter region in tumors is associated with low HACE1 expression (124). Taken together, the epigenetic phenomenon of CpG island hypermethylation in *HACE1* gene promoters, leading to repression and silencing of expression, is an important contributor to oncogenesis.

There are three CpG islands in *HACE1* gene regions, including CpG-88, CpG-177, and CpG-29. The hypermethylation of CpG29 and CpG177 islands upstream of the *HACE1* transcription start site (TSS) is associated with its low expression level was reported in human Wilms tumor (22). Increased methylation of CpG177 is associated with decreased HACE1 expression in tumor tissue compared to normal tissue (30). This has also been shown in other tumors, such as aggressive natural killer cell leukemia (ANKL) (127) and natural killer-cell neoplasms (128). These findings indicate that the expression of HACE1 through demethylation may be a promising therapeutic strategy for malignant tumors.

Previous studies have provided evidence that *HACE1* inactivation in multiple cancers is due to promoter methylation (63). Enhancing HACE1 expression by regulating methylation leads to extensive research on its regulatory mechanism and its tumor-suppressive effect. For example, effective restoration of HACE1 expression in nephroblastoma by the addition of the specific DNA methylation inhibitor 5-azacytidine (5-Aza) (22). NKX6.3 attenuates Helicobacter pylori CagA-induced cellular oxidative stress by inhibiting DNA methyltransferase 1 (DNMT1) and promoting HACE1 expression. The antioxidant activity of NKX6.3 could be eliminated by *HACE1* silencing (129). The histone methyltransferase inhibitor 3-deazaneplanocin A (DZNep) inhibits lymphoma cell growth by promoting HACE1 expression by reducing histone methylation modifications (130). Demethylation of the HACE1 gene promoter has increased HACE1 expression, which inhibited liver cancer cell proliferation by activating OPTN-dependent selective autophagy (90). LINC00161 is a long non-coding RNA closely related to the occurrence and development of hepatocellular carcinoma (HCC), and its expression is upregulated in HCC cells. Inhibition of HACE1 expression by inducing *HACE1* promoter methylation can promote the growth and migration of HCC cells. Knockdown of LINC00161 can reduce the methylation level and increase the expression of HACE1, thereby inhibiting the progression of HCC (131). The expression of MBD3, a demethylation molecule, induced by propofol in a dose-dependent manner, promotes HACE1 protein expression and inhibits lung cancer cell proliferation by activating HACE1-OPTN axis autophagy. However, these therapeutic effects of propofol are eliminated by MBD3 knockout (91). DNA methylation is an epigenetic modification that regulates gene expression that regulates gene expression. It is an important direction for future development and has research value and exploratory significance.

### 7.2 Antioxidants serve as a potential HACE1 activator

**Figure 4:**
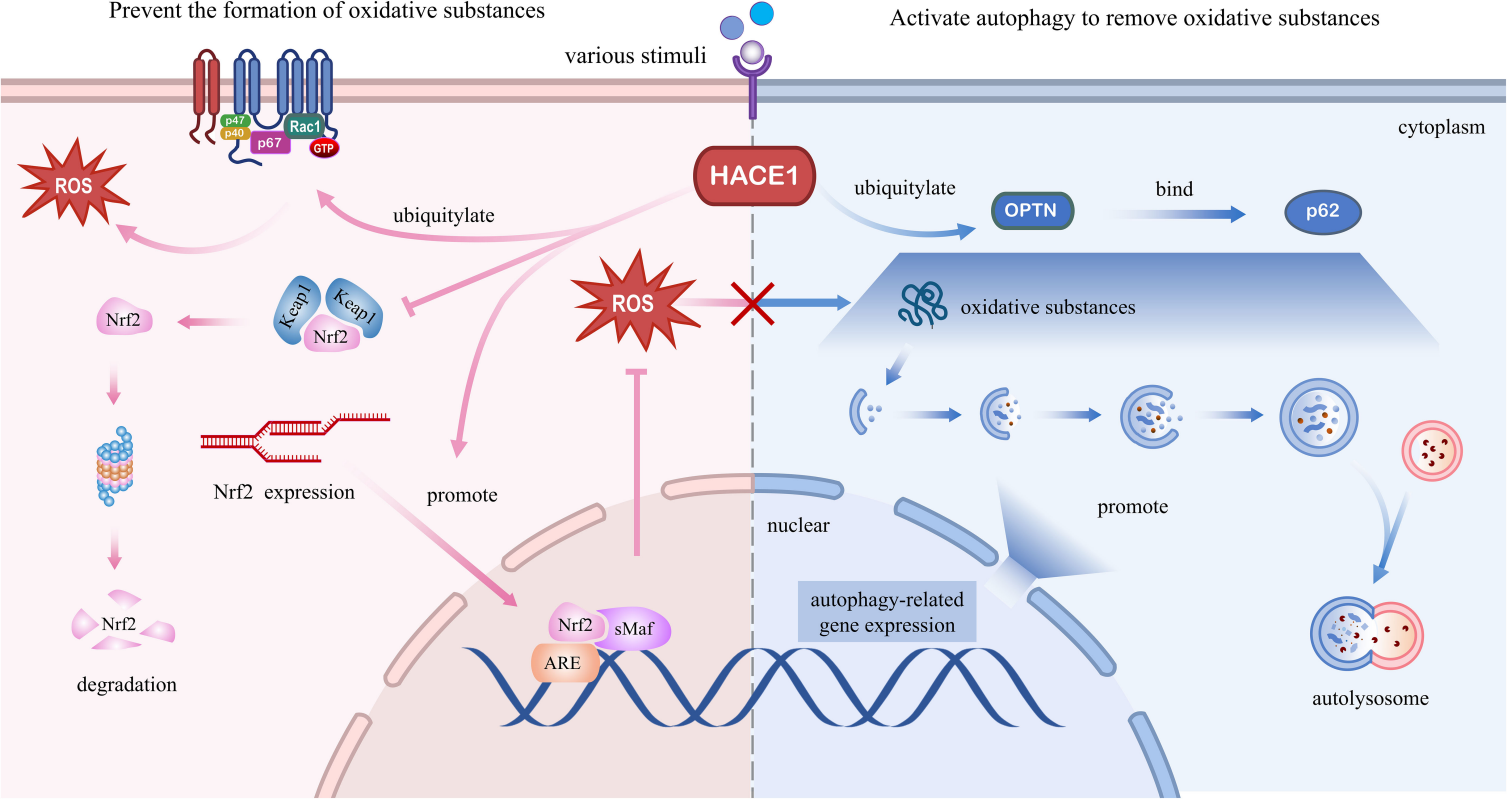
Molecular regulatory mechanisms of HACE1 in degenerative diseases. Left panel: HACE1 prevents the formation of oxidative substances. HACE1 inhibits NADPH oxidase 1-dependent ROS generation by ubiquitinating and degrading Rac1. HACE1 activates the Nrf2/ARE signaling pathway by competitively binding to Nrf2 with Keap1, enhancing the stability of the Nrf2 protein, and promoting the *de novo* synthesis of Nrf2. Right panel: HACE1 activates autophagy to remove oxidative substances. HACE1 activates autophagy by promoting the expression of autophagy-related genes and interacting with the autophagy receptors OPTN and p62.

Gene expression is regulated by multiple pathways, including epigenetic regulation through DNA methylation and histone acetylation (132). We have also discussed in the previous section that HACE1 expression is regulated by the DNA methylation level of the promoter regions. However, HACE1 protein expression is regulated at multiple levels, including protein degradation and gene expression. Previous studies have reported that dimethyl fumarate (DMF), an Nrf2 activator, induces the protein expression of both Nrf2 and HACE1 in a dose-dependent manner, thereby inhibiting the proliferation of T-cell acute lymphoblastic leukemia cells (19). Additionally, Huang and colleagues have found that Magnolol, extracted from the renowned traditional Chinese medicine Magnolia officinalis, exerts its antitumor effects by activating HACE1-OPTN axis-mediated autophagy (20). Finally, the supplementation of the blueberry anthocyanin malvidin 3-glucoside (MG) in colon tissues has been found to increase HACE1 expression and improve the gut microbiota composition (21). The prevalence of degenerative diseases in recent years has triggered extensive research into their pathomechanisms and treatment. At the same time, growing evidence suggests that HACE1 also plays a crucial role in degenerative diseases. HACE1 is an important regulator of cell survival, it maintains cellular homeostasis by activating cellular autophagy and the antioxidant defense system, and its biological mechanism is shown in **Figure 4**. Although the mechanism of action of HACE1 has been well studied, unfortunately, there are no recognized activators of targeted therapy. Therefore, a promising research focus to develop specific and potent activators of HACE1 is an urgent research problem that needs to be addressed.

## 8 Conclusions and perspectives

**Figure 5:**
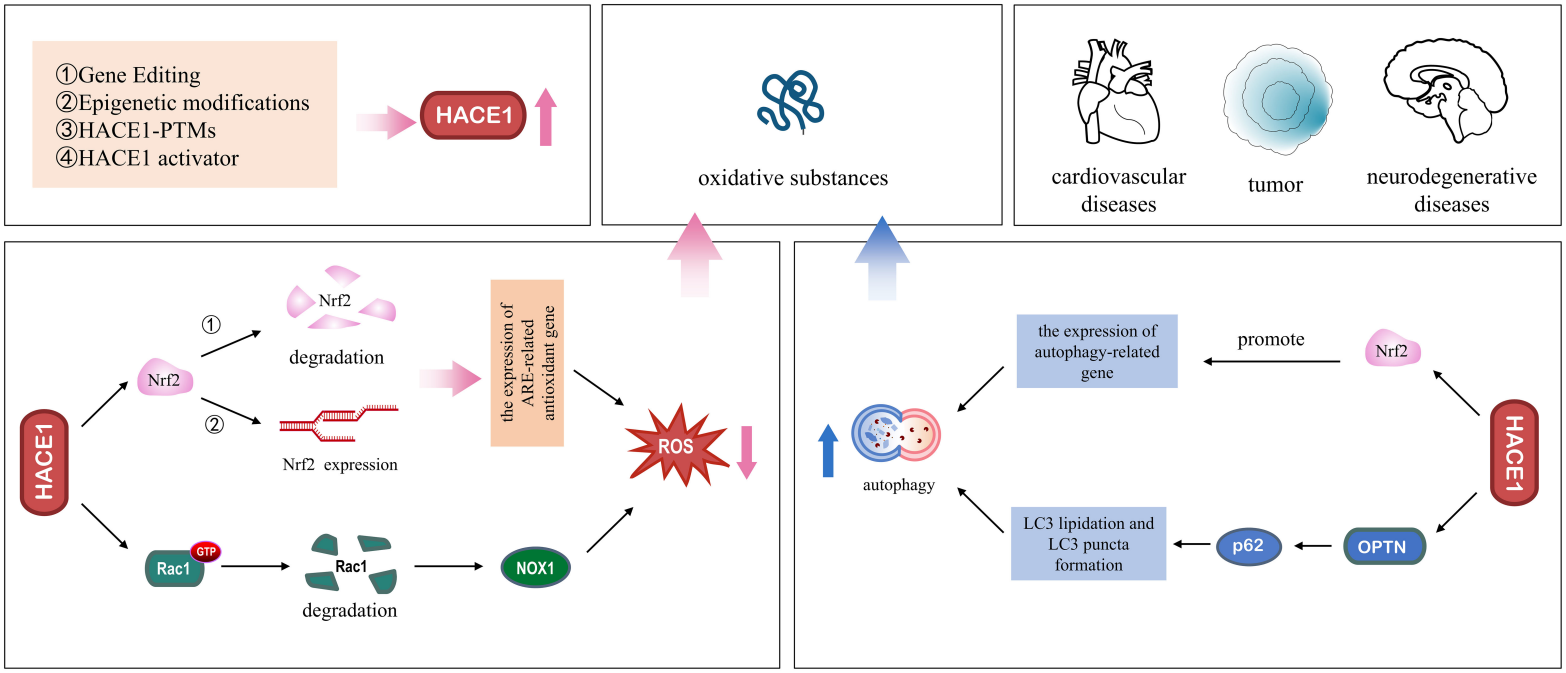
Schematic diagram depicting the role of HACE1 in degenerative diseases.

With the intensification of global aging, the incidence of various age-related degenerative neurological and cardiovascular diseases continues to increase (133). Although the pathogenesis of aging diseases is complex and still unclear, oxidative stress and impaired autophagy have been shown to be key pathogenic mechanisms in these diseases (134). In this review, we provide an overview of oxidative stress and autophagy and summarize the literature related to HACE1, discuss the mechanisms by which HACE1 regulates oxidative stress and autophagy, and highlight its potential therapeutic role in degenerative diseases (**Figure 5**). HACE1 has been identified as an oxidative stress response gene that upregulates cellular antioxidant defenses by targeting Nrf2 and Rac1, reducing the source of oxidative substances. In addition, HACE1 activates autophagy by ubiquitinating selective autophagy receptors OPTN and p62, increasing the degradation of oxidative species. It is well known that the continuous accumulation of non-degradable highly oxidized proteins leading to protein aggregate formation is an important hallmark of aging (135). In addition, the changes of HACE1 content in the serum of patients with heart failure imply its prognostic significance (53).

The two main systems for protein degradation in mammalian cells are ubiquitination-dependent proteasomal degradation and autophagy. HACE1 is an important E3 ubiquitin ligase that acts as a link between the ubiquitination and autophagy machineries. The most common consequence of protein ubiquitination is the subsequent proteasomal degradation of target proteins. When the proteasome is overloaded or its function is inhibited, misfolded proteins can accumulate and form toxic aggregates. Autophagy is an important alternative mechanism for clearing damaged organelles and toxic proteins, especially when oxidative stress causes excessive production of irreversibly oxidized biomolecules. It has already been found that HACE1 is crucial for cell survival, embryonic development, and organ formation. HACE1 maintains cell viability by activating selective autophagy to remove the damaged cell components. Mechanistically, HACE1 scavenges oxidative stress-induced misfolded protein accumulation by activating selective autophagy via ubiquitination of autophagy-associated substrates.

Although Rotblat et al. previously measured HACE1 protein levels in human striatum tissue. Unfortunately, they did not establish a statistical correlation between HACE1 expression and age (45). It seems fortunate that the relationship between HACE1 expression and age has been found in PD mice (67). Based on these reports, we speculate that HACE1 expression would decline with age and exacerbate the onset and progression of degenerative diseases. HACE1, an E3 ubiquitin ligase, is frequently inactivated and has been shown to be a putative tumor suppressor for a variety of malignancies. Therefore, restoring ubiquitination function by adding intracellular free HACE1 protein seems to be a good therapeutic option. However, there are many challenges to this treatment strategy. Specifically, because specific target proteins are numerous and incompletely defined, upregulation of HACE1 E3 ligase activity may lead to degradation or functional changes in other substrate proteins. Additionally, HACE1 ubiquitin ligase activity is disturbed by many factors, resulting in poor stability and difficult delivery.

Ischemia/reperfusion injury is defined as the restoration of blood flow to tissues or organs after transient ischemia, resulting in increased cellular dysfunction and structural damage, which is more severe than ischemia alone (136). The core pathological mechanism is the sudden influx of a large number of oxygen molecules during reperfusion, which triggers the explosive generation of ROS, leading to lipid peroxidation, protein oxidation, and DNA damage (137). In the ischemia-reperfusion injury model, HACE1 has been demonstrated to stabilize mitochondrial function and reduce oxidative stress by activating the Nrf2 signaling pathway, thereby alleviating cellular oxidative damage. It has been evidenced that Nrf2 could regulate the expression of ferroptosis-related proteins, thereby inhibiting the occurrence and development of ferroptosis (138). The study found that HACE1 prevented mitochondrial damage and ferroptosis by targeting Nrf2 in an Ang II-induced mouse model of heart failure, ultimately alleviating cardiac fibrosis and cardiac dysfunction (53). Therefore, the regulatory capacity of HACE1 in ferroptosis has begun to attract attention.

Herein, we sought to use HACE1 as a therapeutic target to explore its protective role and mechanism in aging diseases. However, it is important to note the paradoxical phenomenon of oxidative stress and autophagy in tumors. Nrf2 plays a role in suppressing cancer by protecting normal cells from oxidative stress by activating downstream antioxidant genes. However, the abnormal expression of Nrf2 in tumor cells can also help cancer cells resist oxidative damage and hinder the effect of radiotherapy and chemotherapy (139). It has been found that Nrf2 activation by HACE1 in glioma cells leads to an enhanced malignant phenotype and decreased radiosensitivity (52). Autophagy maintains normal cellular homeostasis in early tumor stages by removing damaged proteins and organelles. However, during tumor development, autophagy provides energy and material support for tumor cells by decomposing intracellular substances and helps tumor cells survive in the unfavorable microenvironment (140). In addition, contrary to that activating autophagy, it was recently reported that overexpression of HACE1 in HT29 cells with HMBOX1 knockdown promotes ubiquitination and degradation of ATG5 K63 to inhibit autophagy and reduce 5-FU resistance in colorectal cancer (141).

Recent research has shown that HACE1 expression is influenced by pharmacological and genetic strategies and plays a potential therapeutic role in age-related diseases such as neurodegeneration, cardiovascular disease, and cancer. Gene editing or vector-mediated approaches can compensate for HACE1 protein, restore its missing protein function, regulate the level of intracellular oxidative substances and affect the development of diseases. Furthermore, epigenetic regulation, including histone modification and DNA methylation, has been shown to regulate HACE1 expression, thereby affecting tumor cell growth. In conclusion, gene therapy and epigenetic regulation to regulate HACE1 protein expression is a feasible therapeutic strategy. In addition, Certain natural antioxidants have been reported to promote the expression of HACE1, which may be involved in cell survival mechanisms including oxidative stress and autophagy regulation through the ubiquitination pathway. Therefore, understanding the fine molecular regulation of oxidative stress and autophagy by HACE1, as well as the close relationship between HACE1 and various cytokines, could provide valuable information. This information may be useful in the future to improve the treatment of age-related diseases and develop new selective therapies.
